# Development of an Original Three-Dimensional Computed Tomography Scan Method and Imaging Process for Surgical Support of the Anterior Cruciate Ligament

**DOI:** 10.7759/cureus.59307

**Published:** 2024-04-29

**Authors:** Minoru Ishifuro, Nobukiyo Yoshida, Kazushi Yokomachi, Chikao Fujioka, Nobuo Adachi

**Affiliations:** 1 Department of Radiological Technology, Niigata University of Health and Welfare, Niigata, JPN; 2 Department of Clinical Support, Hiroshima University Hospital, Hiroshima, JPN; 3 Department of Orthopedic Surgery, Graduate School of Biomedical and Health Sciences, Hiroshima University, Hiroshima, JPN

**Keywords:** surgical navigation system, knee joint flexion, bone tunnel, anterior cruciate ligament reconstruction, three-dimensional ct

## Abstract

Three-dimensional computed tomography (3D CT) scan images are useful as they can provide information essential for surgical support, particularly in orthopedic surgery. In the case of anterior cruciate ligament (ACL) reconstruction, a 3D CT scan is important in preoperative simulation. Furthermore, it is associated with a reduced risk of revision surgery because the angle of the foramen magnum changes with the femoral muscle mass. However, the CT scan system geometry has several limitations. For example, the patient’s posture is limited during the procedure. Herein, we report an original CT scan method and 3D imaging process for surgical support of the ACL.

## Introduction

In diagnostic computed tomography (CT) scans, three-dimensional (3D) images can provide information on the target area or disease. Hence, they are useful in various procedures and treatments. The history of 3D imaging has been short since the delivery of 3D images from CT. However, the field of medicine has achieved rapid progression. In particular, 3D images can relay indispensable information for surgical support. In the field of orthopedics, 3D imaging is important for detecting fractures and dislocations. In soft tissue diseases, 3D images can also be used to determine whether conservative treatment or surgical procedures are required. Herein, we report an original CT scan method and 3D imaging process for surgical support of the anterior cruciate ligament (ACL).

CT scanning is advantageous and can replace 3D imaging. 3D images used for ACL reconstruction must be obtained in the same position as the surgical position. However, CT scan systems replace the imaging data acquired within a limited gantry size with 3D images. Hence, it can be disadvantageous due to limited positioning. In addition, positioning has a significant impact on evaluation in the field of orthopedic surgery. In particular, in ACL reconstruction, the knee joint on the affected side is in the most flexed position in the supine position. Therefore, imaging cannot be performed in this position. Herein, we report the usefulness of a newly devised imaging processing method for surgical planning.

## Case presentation

CT scan dose and image quality

The current study retrospectively analyzed Japanese patients who visited Hiroshima University Hospital from October 2011 to March 2019 and who underwent surgery for ACL injury. The Research Ethics Committee of Hiroshima University Hospital approved the study protocols (no. 446, dated October 12, 2011). The need for a written informed consent was waived.

The following CT scan systems were used: GE (GE Healthcare Japan, Milwaukee, USA) and Canon (Canon, Tokyo, Japan). The 3D image processing Virtual Place (Canon, Tokyo, Japan) was used for ACL 3D image acquisition. The GE CT scan systems were as follows: 120 kVp, auto mA, 0.5 s/rot. The reconstruction parameters were as follows: software or standard, slice: 1.25 mm, field of view: 20 cm. The Canon CT systems were as follows: 120 kVp, auto mA, 0.5 s/rot. The reconstruction parameters were as follows: FC01 or FC08-H, slice: 1.0 mm, field of view: 20 cm. The dose of each CT device was 20-32 mGy in the preoperative maximum knee flexion position and 10-23 mGy in the postoperative 30º flexion position. These imaging conditions are for 3D images. Thus, a reconstructed slice thickness of approximately 1 mm was selected to reduce the amount of noise. The smooth function was used for the image filter function (GE soft function, Canon FC01). In addition, noise reduction processing was performed on the workstation to prevent affecting image quality.

CT scan position

The supine position with the affected knee joint side at maximum flexion is the basic surgical position in ACL injury reconstruction [1-3]. However, it is challenging to position the joint at the maximally flexed position in the center of the CT scan gantry. Moreover, with this position, the patient cannot enter the gantry. To address this issue, the prone position is considered as the affected knee joint can be positioned at the center of the gantry in the same maximum flexion as that during surgery. Moreover, a CT scan can be performed with the affected knee joint positioned at the center of the gantry [4].

Presurgical CT Scan

**Figure 1:**
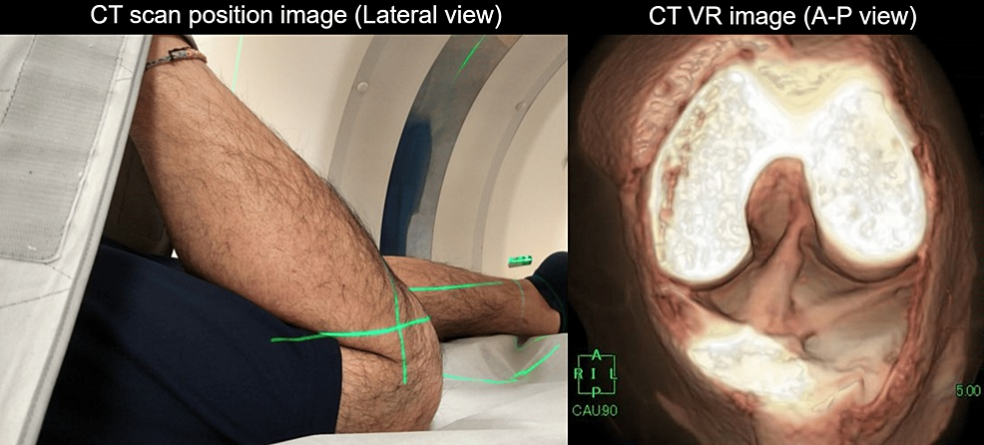
CT imaging positioning and CT volume rendering (VR) image of the knee joint in maximum flexion, equivalent to anterior cruciate ligament reconstruction surgery. Generally, anterior cruciate ligament reconstruction is performed while the patient is in the supine position with maximum flexion of the knee joint on the affected side. However, It was challenging to obtain 3D images for preoperative CT scan simulation in this position because the body hit the gantry. Thus, the patient should be in the prone position to maintain the maximum flexion angle of the knee joint. A-P, anterior-posterior.

During a CT scan, patients could be placed in the prone position with maximum knee flexion at the center of the gantry, which is similar to that in ACL reconstruction. In addition, stability could be maintained by foxing the band (Figure 1).

**Figure 2:**
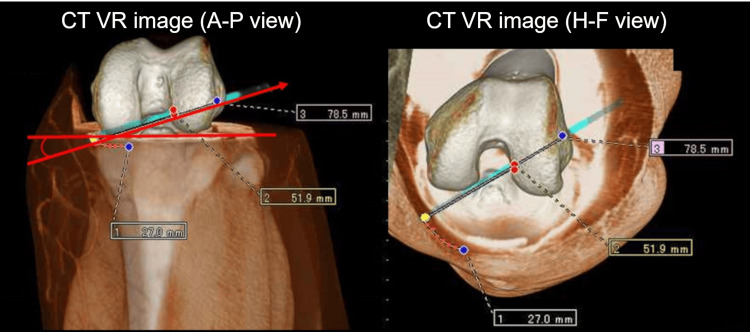
Image of a preoperative CT scan simulation. In ACL reconstruction, the medial and lateral condyles of the knee were aligned to identify the straight side. Then, they were returned to the front at 90º, and the tunnel installation on the straight front 3D was planned. The angle of incidence and distance was measured. VR, volume rendering; A-P, anterior-posterior; H-F, head-foot.

In ACL reconstruction, the medial and lateral condyles of the knee were aligned to identify the straight side. Then, they were returned to the front at 90°, and the tunnel installation on the straight front 3D was planned. The angle of incidence and distance was measured (Figure 2).

CT Scan Immediately After Surgery

**Figure 3:**
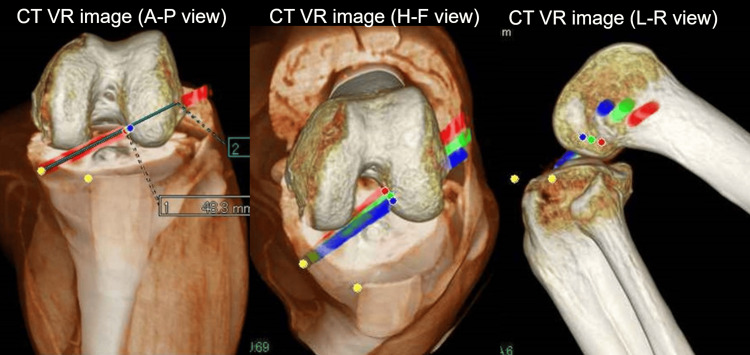
Three-dimensional image obtained by simulating several tunnels for the placement of reconstructive ligaments in anterior cruciate ligament reconstruction from 3D CT scan images. In patients with an average muscle mass, preoperative planning was performed to achieve the green line. However, patients with a large thigh muscle mass had a lower maximum flexion angle, resulting in a red line for the bone tunnel position. By contrast, patients with a small muscle mass could have a higher maximum flexion angle resulting in the blue line. Therefore, the maximum flexion angle of the knee joint differed based on muscle mass. Furthermore, the position of the bone tunnel was misaligned; thus, a CT scan simulation with 3D images was required. VR, volume rendering; A-P, anterior-posterior; H-F, head-foot; L-R, left-right.

The patients wore a knee brace immediately after surgery and for several days thereafter to promote recovery. A 3D CT scan was performed to validate the position of the reconstructed ligament. To maintain a stable state of recovery, an auxiliary tool was used during imaging to perform procedures including a 3D CT scan. Figure 3 shows a prereconstructed 3D CT scan image of a partial ACL tear taken in the prone position. 3D images were obtained by simulating several tunnels during the placement of reconstructive ligaments in ACL reconstruction from 3D CT scan images.

CT Scan Six Months and One Year After Surgery

**Figure 4:**
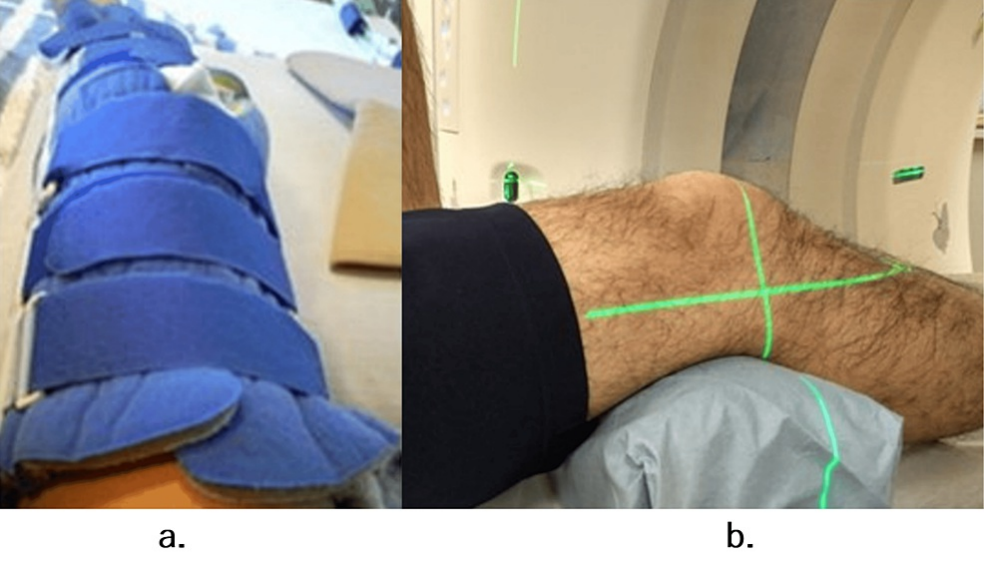
Postoperative CT scan imaging. Follow-up CT after ACL surgery is shown. Remove the knee brace for fixing the knee (a) and maintain the position below the knee joint using an auxiliary tool to maintain the same positioning as that for fixation (b).

A 3D CT scan was performed at 30º knee flexion in the supine position six months and one year after surgery (Figure 4).

**Figure 5:**
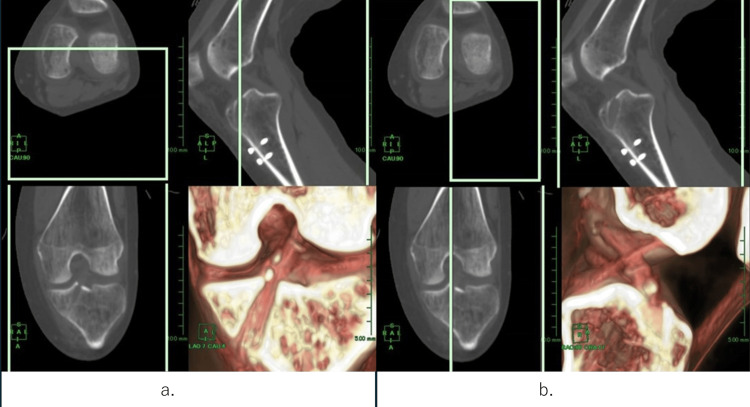
Follow-up 3D images. The anterior cruciate ligament (ACL) is observed from the front (a); both ACL and posterior cruciate ligaments are observed from the lateral side (b). Assessment was performed by cutting out the cross-section indicated by the multi-planar reconstruction, using the clip function to clip the obstructive tissue on the image, and observing the reconstructed ACL in multiple directions.

This image showed the performance of the 3D CT scan six months after surgery (Figure 5).

The section indicated by the multiplanar reconstruction was cut out, and the clip function was used to clip the obstructive tissue on the image. Moreover, the reconstructed ACL was observed from multiple directions.

## Discussion

The patients were placed in the supine position with maximum knee joint flexion on the affected side during preoperative ACL diagnosis using 3D imaging and CT scan positioning for surgical planning. The knee joint was evaluated at 30º during preoperative evaluation.

Axial images taken using this technique have a wide frontal view of the knee joint cavity and a clear view of the ACL. In the extended position, the knee joint cavity is narrower, and the ligament is generally observed in the lateral magnetic resonance imaging image. However, details of the injury cannot be completely obtained because the image is two-dimensional. The current imaging technique facilitates an easy assessment of the ACL on a 3D image. Hence, it can replace magnetic resonance imaging due to its ability to simultaneously acquire image information for ACL diagnosis and surgical navigation [5-9].

In preoperative planning, when drilling a bone hole in the distal condyle of the femur, which is the basic surgical method, in patients with a large femoral muscle mass, the angle of the bone hole becomes shallow, and reoperation may be required. Osteotomy placement for ACL reconstruction has a statistically significant base angle [10]. Preoperative planning using 3D CT scan images can plan the installation angle of the bone tunnel in advance. Moreover, preoperative simulation using 3D CT scan images is also important to prevent re-tears [5,11,12].

In summary, in ACL reconstruction, the angle of the bone tunnel changes with the femoral muscle mass, and 3D CT scan images can provide important information for preoperative diagnosis. Therefore, 3D CT scan images can be used to calculate the flexion angle by taking muscle mass into consideration, which may reduce the risk of revision surgery. The current study had several limitations. That is, the preoperative and postoperative six-month imaging postures differed because of the retrospective nature of the study. Furthermore, the knee flexion angles at the preoperative, immediate postoperative, and six-month follow-up periods varied, and the 3D CT scan images could not be compared with the same knee flexion angle for all patients.

## Conclusions

The prone position during CT scanning allows the joint to be placed in the center of the gantry in a maximally flexed position. 3D CT scanning was useful for ACL reconstruction because it can facilitate the simultaneous evaluation of the ligament and bone. Thus, it can provide the best imaging information for preoperative simulation.
